# Bragg-Mirror-Assisted High-Contrast Plasmonic Interferometers: Concept and Potential in Terahertz Sensing

**DOI:** 10.3390/nano10071385

**Published:** 2020-07-16

**Authors:** Youqiao Ma, Jinhua Li, Zhanghua Han, Hiroshi Maeda, Yuan Ma

**Affiliations:** 1 School of Physics and Optoelectronic Engineering, Nanjing University of Information Science and Technology, Nanjing 210044, China; lijinhua@nuist.edu.cn; 2 Shandong Key Laboratory of Optics and Photonic Devices, School of Physics and Electronics, Shandong Normal University, Jinan 250358, China; zhan@sdnu.edu.cn; 3 Department of Information and Communication Engineering, Fukuoka Institute of Technology, Fukuoka 811-0295, Japan; hiroshi@fit.ac.jp; 4 Department of Electrical and Computer Engineering, Dalhousie University, Halifax, NS B3J 2X4, Canada; yuan.ma@dal.ca

**Keywords:** plasmonics, terahertz, interferometers, refractive index sensors

## Abstract

A Bragg-mirror-assisted terahertz (THz) high-contrast and broadband plasmonic interferometer is proposed and theoretically investigated for potential sensing applications. The central microslit couples the incident THz wave into unidirectional surface plasmon polaritons (SPPs) waves travelling to the bilateral Bragg gratings, where they are totally reflected over a wide wavelength range back towards the microslit. The properties of interference between the SPPs waves and transmitted THz wave are highly dependent on the surrounding material, offering a flexible approach for the realization of refractive index (RI) detection. The systematic study reveals that the proposed interferometric sensor possesses wavelength sensitivity as high as 167 μm RIU^−1^ (RIU: RI unit). More importantly, based on the intensity interrogation method, an ultrahigh Figure-of-Merit (FoM) of 18,750% RIU^−1^, surpassing that of previous plasmonic sensors, is obtained due to the high-contrast of interference pattern. The results also demonstrated that the proposed sensors are also quite robust against the oblique illumination. It is foreseen the proposed configuration may open up new horizons in developing THz plasmonic sensing platforms and next-generation integrated THz circuits.

## 1. Introduction

Over the past several decades we have witnessed a rapidly growing development in the optical sensors for applications of biological and chemical detection [1]. Numerous optical sensors, such as the Mach–Zehnder interferometers [2], optical fibers [3], surface enhanced Raman scattering (SERS) [4] and surface plasmon polaritons (SPPs) [5], have been intensively proposed and developed. Among these sensing schemes, the SPPs technique is becoming one of the most attractive approaches for the ultrasensitive biosensing in recent years due to their distinct merits for on-chip integration [5]. SPPs, which are the electromagnetic (EM) waves coupled to electron oscillations and propagating along the interface between a dielectric and a metal, are highly localized in the vicinity of the metal surface, making them extremely sensitive to the variations of the surrounding dielectric environment. Therefore, the SPPs structures have been widely used in various real-time and label-free sensing applications [6]. Furthermore, the SPPs have characteristics of strong mode confinement and field enhancement, which show a great potential in constructing the large-scale sensing arrays with independent functional elements [7].

Currently, a well-developed plasmonic sensor is based on the prism coupling configuration, which is known as the Kretschmann or Otto configuration [8]. Although this type of SPPs sensors possesses a high sensitivity, they suffer from the disadvantages of their bulky sizes. To overcome this limitation, researchers have proposed a variety of highly integrated structures, such as the subwavelength periodic configurations, the nanoparticles and the nano-interferometers [9,10,11]. For example, a nanoplasmonic biosensor composed of the gold nanohole arrays supporting the extraordinary optical transmission (EOT) was developed to monitor the live cell cytokine secretion in a label-free configuration, which achieves a compact chip area of 100 μm × 100 μm and experimentally obtained a sensitivity up to 1 ng/mL [12]. N. M. Y. Zhang et al. recently developed a compact biosensor with gold nanoparticles integrated with a microfiber, which delivers the promising localized SPPs (LSPPs) properties and ultralow detection limit of cholesterol molecules of 5 aM [13]. X. Zheng et al. also reported a plasmonic interferometer array sensor, which can be imaged by a miniaturized microscope system coupled with a smart phone to realize a portable healthcare device, exhibiting a superior sensing resolution of 1.63 × 10^−6^ refractive index unit (RIU) [14]. Although the SPPs biosensing technology has developed within the optical frequency, the light wave energy is relatively high, or in other words, a photoionization reaction usually takes place during the detection process, which not only affects the activity of the biomolecules, but also makes the ions react with each other, leading to the more complicated results and unstable detection [15]. Meanwhile, most of the noble metals have inherent ohmic losses that are inevitable in the optical window, especially when the SPPs mode size is compressed to the sub-wavelength level, the mode loss will be further increased, which in turn results in a low sensitivity and resolution [16].

In order to address these limitations, the researchers started to focus on the infrared, terahertz (THz) and microwave frequency bands [17]. Owning to the low radiation energy, large instantaneous bandwidth and rich spectrum information, the THz technology has rapidly attracted a wide interest [18]. More importantly, the vibration/rotation frequency of many biomolecules locates in the THz band, which potentially offer a feasible way to build the standard “fingerprint” library [19]. Therefore, the combination of the THz detection and SPPs technologies is suggested to promote the development of the biosensors. Among them, the research of metamaterial structures has been a hot topic in recent years [20]. For example, a THz metamaterial biosensor based on the split-ring resonators was theoretically and experimentally demonstrated for enhanced fingerprint detection of lactose [21]. Although the metamaterials possess unique physical properties that most materials in nature do not have, the shortcomings of the metamaterial sensors are also obvious, such as the structural complexity and difficulty of fabrication.

The biosensors mentioned above are typically based on the metal materials. However, the free electron density of metal is very high, which makes it difficult to tune the dielectric constant [22]. Therefore, the metallic structures need to be specially designed to meet the specific requirements, which leads to the poor flexibility of the structure. The researchers thus have to seek the better SPPs excitation materials. For example, it has been found that doped semiconductors and graphene exhibit dielectric properties in the THz window, which are very similar to those of metals in the optical windows, and the characteristics of SPPs can be controlled by changing the carrier concentration, which is a huge advantage that the traditional metal materials do not have [23]. In recent years, tunable semiconductor-based biosensors are getting more and more attention [24,25]. For example, F. B. Barho et al. proposed and experimentally demonstrated a one-dimensional (1D) periodic all-semiconductor (highly doped InAsSb gratings on GaSb substrate) biosensing platform with a sensitivity of 900 nm/RIU [24]. However, the sensing performance for the 1D grating structures is highly affected by the polarization state of the incident light. To address the polarization-sensitive problem, W. D. Xu et al. also proposed a three-dimensional (3D) metamaterial THz biosensor by integrating a monolayer graphene and experimentally showed its ultrahigh sensitivity through detecting trace amount of chlorpyrifos methyl down to 0.2 ng [25]. The periodic structure-based biosensors can easily realize the on-chip integration, however, they suffer from their narrow-band resonant linewidths, or in other words, their sensitivities decrease significantly when the response spectra are away from the resonant wavelengths [26]. To address this spectroscopic issue, we recently proposed and studied several semiconductor-based interferometers based on the waveguide-coupled and slit-groove patterned structures [27,28]. However, it is still believed that finding new realizable schemes to further enhance the interferometric intensities and spectral contrast remains highly desired.

Herein, we propose and systematically investigate a robust SPPs interferometer for the ultrasensitive THz sensing by introducing the SPPs band-gap mirrors to significantly enhance the reflected intensities of interferometric SPPs waves. The proposed structure is formed by patterning a microslit flanked by two Bragg gratings in a semiconductor layer. The interference between the SPPs waves scattered by Bragg gratings and transmitted THz waves leads to a strong modulation in the transmitted intensity. The interference pattern will be shifted when the permittivity of surrounding material is varied. The calculations reveal that the proposed interferometric sensor exhibits an refractive index (RI) sensitivity as high as 160 μm/RIU and a high figure-of-merit (FoM) of 18,750% RIU^−1^ based on the intensity interrogation method. This paper is organized as follows: In Section 2, the basic considerations and design principle are introduced. From Section 3 to Section 5, the results are discussed in detail using the finite element method (FEM), including the design of the planar Bragg reflectors, the discussion about the Bragg mirrors-assisted interferometry and the investigation of a RI sensors based on the optimized structural parameters. Finally, the conclusions are discussed in Section 6.

## 2. Basic Considerations

**Figure 1:**
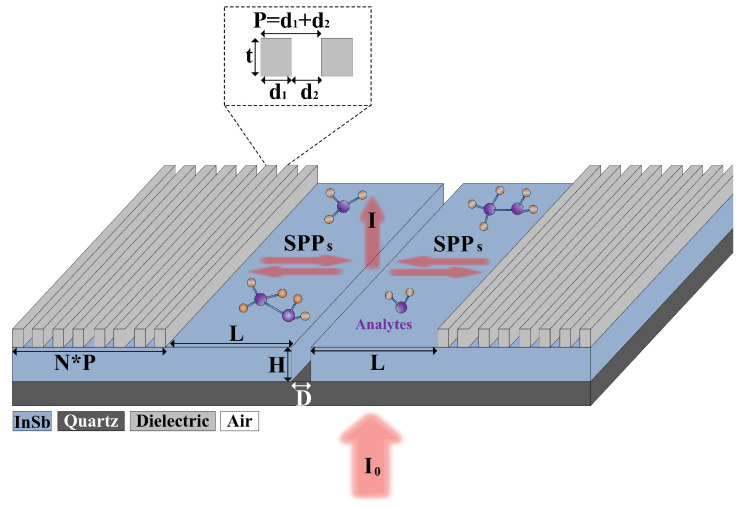
Three-dimensional (3D) view of proposed surface plasmon polaritons (SPPs) interferometer structure, which is composed of a microslit patterned InSb film coated on a quartz substrate. Two identical Bragg gratings are placed on both sides of microslit. Inset: Schematic view of a unit of Bragg grating.

A schematic sketch of the proposed interferometer configuration is shown in Figure 1. The structure consists of a patterned semiconductor film deposited onto a quartz (SiO_2_) substrate, which contains a central microslit (with a width of *D* and a thickness of *H*) flanked by two identical Bragg gratings (with a height and period of *t* and *P*, respectively, and the number of period is denoted as *N*) with a separation length of *L*. The Bragg gratings are periodically modulated by the air and dielectric (the specific dielectric material will be discussed later). All the structural characteristics and coordinate are depicted in the figure. The indium antimonide (InSb) is selected for the semiconductor material because it possesses a large electron density and narrow energy gap. The permittivity of InSb (*ε*_InSb_) is described by the Drude model [29]

(1) $$\varepsilon_{\mathrm{InSb}}=\varepsilon_{\infty }-\frac{Ne^{2}}{\varepsilon_{0}m_{\mathrm{eff}}\left(\omega^{2}+i\gamma \omega \right)}$$

where $\varepsilon_{0}$ and $\varepsilon_{\infty }$ are the vacuum permittivity and high-frequency permittivity, respectively, *ω* is the angular frequency, *γ* is the damping constant, *e* is the electron charge, *m*_eff_ is the effective mass of a free carrier and *N* is the intrinsic carrier density, which can be written as [30]

(2) $$N\left({\mathrm{cm}}^{-3}\right)={\left(2400-T\right)}^{\frac{3}{4}}T^{\frac{3}{2}}\left(2.9\times 10^{11}+7.83\times 10^{7}T\right)\exp\left(\frac{1.5\times 10^{-4}T-0.129}{K_{B}T}\right)$$

where *T* and *K*_B_ are the operating temperature and Boltzmann constant, respectively. In the following discussions, the parameters of the Drude model for InSb are set as $\varepsilon_{\infty }$ = 15.68, *γ* = 0.1p THz and *m*_eff_ = 0.015*m*_e_ (where *m*_e_ is the mass of electron). A finite element method (FEM) was utilized to study the mode/transmission properties, and the perfectly matched layer (PML) was employed at the calculation boundaries to absorb the reflection of the outgoing THz radiations. The convergence tests were also done to assure the meshing and boundaries have no effect on the solutions.

## 3. Design of Planar Bragg Mirrors

To better design the Bragg gratings, it is essential to understand the mode properties for SPP modes existing on a planar multilayer structure, i.e., air-dielectric-InSb, in which the air and InSb are assumed as the semi-infinite superstrate and substrate, respectively, and the thickness of the dielectric layer is known as *t*. Two widely used dielectrics in the Complementary metal–oxide–semiconductor (CMOS) industry were selected for discussion, which are silicon (Si) and SiO_2_, as they have negligible losses within the THz frequency of interest [31]. In addition, their refractive indices are almost frequency (temperature) independent within a frequency (temperature) ranges from 0.8 (280 K) to 1.5 THz (320 K), thus their refractive indices are set as constants of $n_{Si}$ = 3.4 and $n_{SiO_{2}}$ = 1.95 in the present work [31]. For simplification, we defined the SPPs modes existing on the air-SiO2-InSb waveguide and air-Si-InSb waveguide as SPPs(SiO_2_) and SPPs(Si), respectively.

**Figure 2:**
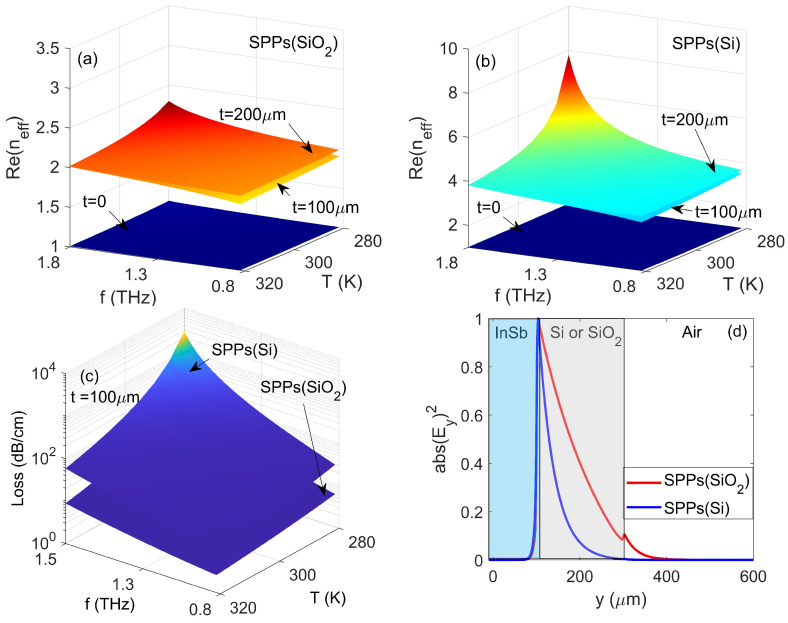
Values of Re(*n*_eff_) as functions of *f* and *T* with different *t* for (**a**) SPPs(SiO_2_) and (**b**) SPPs(Si) modes. (**c**) Dependence of mode losses for SPPs(SiO_2_) and SPPs(Si) modes on parameters of *f* and *T*. (**d**) Normalized electric energy density patterns for SPPs(SiO_2_) and SPPs(Si) modes with parameters of *f* = 1 THz, *T* = 300 K and *t* = 100 μm.

Figure 2a,b shows the dependence of real parts of the mode effective refractive index, i.e., Re(*n*_eff_) for SPPs(SiO_2_) and SPPs(Si) modes on parameters of *T*, *f* and *t*, respectively. As shown in Figure 2a,b, the value of Re(*n*_eff_) increased monotonically with the increase (decrease) of *f* and *t* (*T*). Compared to a single air-InSb interface (i.e., *t* = 0), the waveguide with Si cladding exhibited a better Re(*n*_eff_) contrast than that for a waveguide with SiO_2_ cladding. This is physically reasonable due to the fact that $n_{Si}>n_{SiO_{2}}$. Therefore, the resulting Bragg frequency gap was wider if Si and air were alternately stacked. However, a higher value of Re(*n*_eff_) basically corresponded to a stronger mode confinement and a larger propagation loss. For example, Figure 2c depicts the mode losses for SPPs(SiO_2_) and SPPs(Si) modes as functions of *f* and *T* with *t* = 100 μm. As expected, it is found that the waveguide with Si cladding possessed a higher loss. To examine this in detail one-dimensional (1D) mode patterns for SPPs(SiO_2_) and SPPs(Si) modes are plotted in Figure 2d with parameters of *f* = 1 THz, *T* = 300 K and *t* = 100 μm. It is clear that the mode confinement for SPPs(Si) mode is stronger, resulting in a larger portion of power distributed in the vicinity of InSb region thus a larger conductor dissipation. Therefore, by balancing the trade-off between the mode confinement, the Re(*n*_eff_) contrast and mode loss, the material of SiO_2_ will be selected for constructing the Bragg gratings, and the height of the SiO_2_ layer is set as *t* = 100 μm.

**Figure 3:**
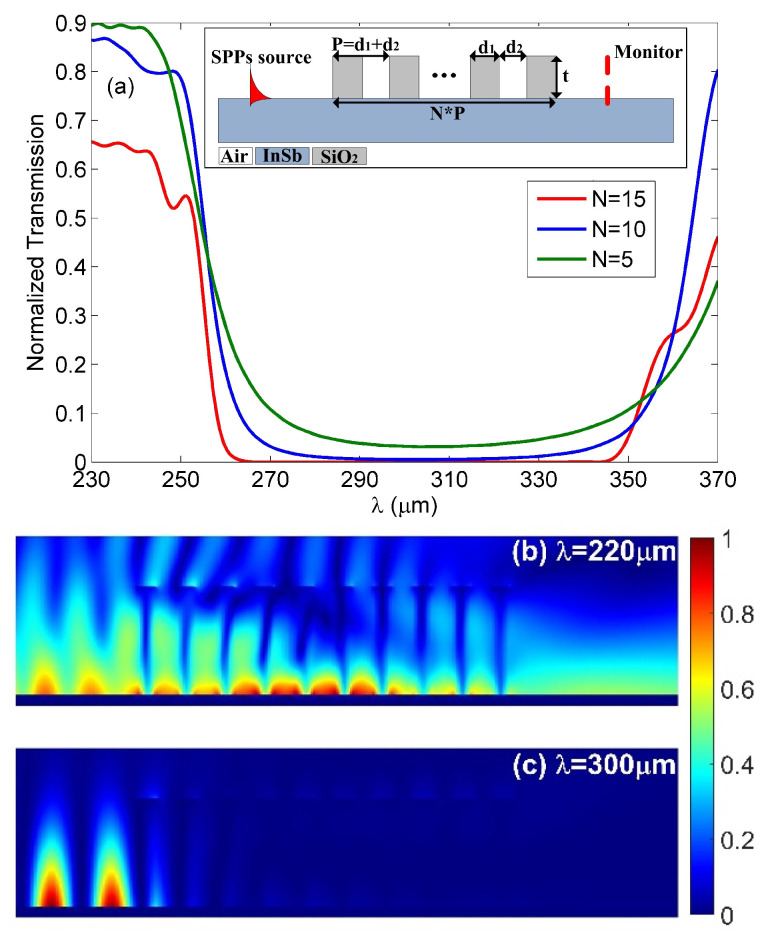
(**a**) Normalized transmission spectra of Bragg gratings with different number of period N. Inset: schematic view of Bragg reflector. Contour profiles of energy density at different incident wavelength of (**b**) *λ* = 250 μm and (**c**) *λ* = 300 μm.

As depicted in Figure 2a, it shows that the air-SiO_2_-InSb waveguide with different height *t* led to the contrast of Re(*n*_eff_), therefore, a periodic change of Re(*n*_eff_) can be produced if SiO_2_ and air are arranged periodically along the direction of SPPs propagation to form the well-known Bragg reflectors (or SPPs band-gaps). The Bragg grating configuration is shown in the inset of Figure 3a with all structural parameters indicated, and a time monitor (i.e., red dotted line) is located at the right side of the Bragg grating to detect the transmitted power of SPPs wave. Based on the Bragg condition, the Bragg wavelength *λ*_Bragg_ can be expressed as

(3) λBragg=2d1Re(neff(t=100μm))+2d2Re(neff(t=0))

where $n_{\mathrm{eff}\left(t=100\mu m\right)}$ and $n_{\mathrm{eff}\left(t=0\right)}$ represent the values of Re(*n*_eff_) for the air-SiO2-InSb waveguide with *t* = 100 μm and *t* = 0, respectively. According to Equation (4), we could realize the Bragg scattering around *λ*_Bragg_ = 300 μm (i.e., *f* = 1 THz) by choosing *d*_1_ = 55 μm and *d*_2_ = 50 μm. The normalized transmission spectra of SPPs wave propagating though the Bragg grating structure with a different number of period *N* is shown in Figure 3a. The parameters used were *d*_1_ = 55 μm, *d*_2_ = 50 μm, *T* = 300 K and *t* = 100 μm. The normalization was achieved with the transmitted intensity through the structure with Bragg grating divided by that for a structure without Bragg grating. As shown in Figure 3a, there were obvious SPPs band gaps around *λ*_Bragg_ = 300 μm when a finite number of periods (*N*) was considered. With an increase of *N*, the width of band gap remained almost unchanged, while the transmission efficiency decreased. The reason is that a larger *N* means a longer propagation length along the SiO_2_-InSb interface and hence a larger dissipation loss. To further verify the SPPs band gaps, the normalized THz energy density patterns of the structure at wavelengths of 250 μm (out of the band-gap) and 300 μm (within the band-gap) were investigated and summarized in Figure 3b,c, respectively. It is clearly found that the incident THz radiation can propagate through the Bragg grating at *λ* = 250 μm, which was outside the SPPs band-gap, while when the incident wavelength (i.e., *λ* = 300 μm) was located within the SPPs band-gap, the SPPs wave was almost reflected and the transmission was forbidden. The results were in good agreement with the transmission spectrum shown in Figure 3a.

## 4. Discussion of Bragg Mirrors-Assisted SPPs Interferometer

As depicted in Figure 1, when a TM-polarized THz wave (with a magnetic vector parallel to the slit) normally launches the microslit from the bottom side, it partially scattered and coupled into the bidirectional SPPs waves on the upper surface propagating towards the bilateral Bragg gratings. They then propagated back towards the central microslit due to the Bragg reflection with an additional phase given by *φ*_add_ = 2*k*_SPPs_ *L* + *φ*_1_, where *φ*_1_ represents the total phase occurred during the scattering events at the slit and Bragg gratings, and the wave-vector of the SPPs mode *k*_SPPs_ can be written as

(4) kSPPs=2πfcnd2Re(εInSb)nd2+Re(εInSb)

where *c* and *f* are the speed of light in vacuum and frequency of incident THz wave, respectively, Re(*ε*_InSb_) is the real part of permittivity of InSb, *n*_d_ is the refractive index of the surrounding material on the upper surface. At the slit, the phase delayed SPPs waves interfere with the component of the THz wave directly transmitted from the slit. The resulting total transmitted intensity *I* can be given by

(5) $$I\propto {\left|1+2e^{i\left(2k_{\mathrm{SPPs}}L+\varphi_{\mathrm{add}}\right)}\right|}^{2}$$

**Figure 4:**
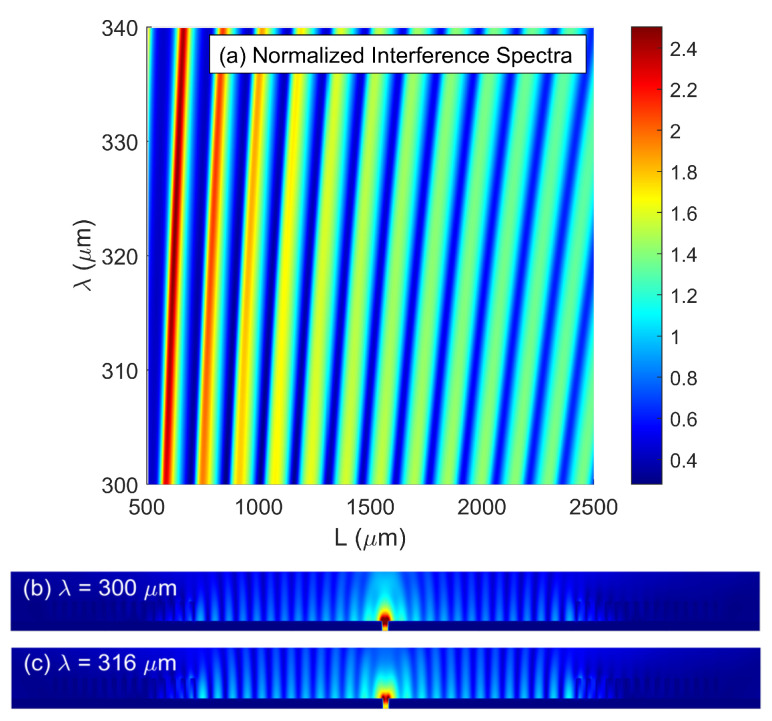
(**a**) Contour plot of normalized transmitted intensity as functions of both separation length *L* and wavelength *λ* for proposed SPPs interferometers. Calculated electric field distributions at (**b**) constructive wavelength of 300 μm and (**c**) destructive wavelengths of 316 μm.

According to Equation (5), the destructive (constructive) interference takes place when the total phase difference is equal to the odd (even) multiples of π. Figure 4a shows a contour plot of normalized transmitted intensity of the proposed SPPs interferometer with air surrounded (i.e., *n*_d_ = 1) versus both the separation length (*L*: 500–2500 μm) and wavelength (*λ*: 300–340 μm) at room temperature (*T* = 300 K). The parameters used in the simulations were *N* = 15, *t* = 100 μm, *d*_1_ = 55 μm, *d*_2_ = 50 μm, *D* = 60 μm and *H* = 300 μm. The normalization was achieved by normalizing the transmitted intensity to that of an isolated microslit structure. In Figure 4a, the obvious spectral oscillation (i.e., interference pattern) was observed. The transmitted intensity gradually became smaller as *L* (*λ*) increased (decreases) because of the increased propagation loss (enhanced material absorption loss). Physically, the suppression (enhancement) of the interference spectra arose from the destructive (constructive) interference between the transmitted THz wave and SPPs waves. One could also find that the number of peaks and valleys increased with the increase of *L* due to the fact that the interference period *P* was inversely proportional to *L,* i.e., *P*∝*λ*^2⁄*L*. Therefore, the interference peaks can be easily tuned narrower by increasing the path length *L*, which are especially desired for a sensor design to improve its accuracy and resolution of detection. However, a longer length *L* gives rise to a higher propagation loss, which in turn lowers the interference contrast. On the other hand, one can adjust the dimensions of microslit to control the intensity of scattered THz radiation, which will couple into the SPPs waves. Hence, the parameters of the microslit (i.e., *D* and *H*) can be approximately designed to maximize the intensities of two interfering SPPs waves in the spectral range of interest. In order to maximize the excitation efficiency of SPPs waves, the physical parameters of the slit were chosen as *D* = 60 μm and *H* = 300 μm [28]. Figure 4b,c shows the simulated electric field distributions at positions of interference peak (*λ* = 300 μm) and valley (*λ* = 316 μm) with a separation length of *L* = 2000 μm, respectively. Due to the constructive and destructive interference, the strong THz wave transmission and suppression were clearly visualized at the output of the slit, respectively.

**Figure 5:**
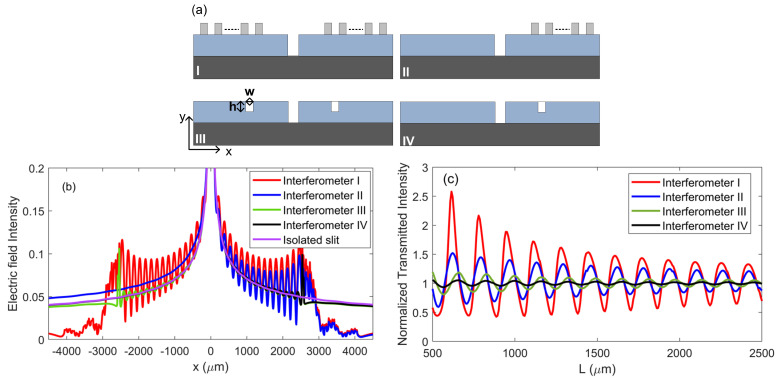
(**a**) Schematic views of four different interferometers with bilateral Bragg gratings (I), one Bragg grating (II), bilateral grooves (III) and one groove (IV). (**b**) Electric field intensity distributions for four interferometers and isolated slit along upper surface of semiconductor. (**c**) Normalized transmitted intensity versus path length L for four different interferometers.

Moreover, to demonstrate the merits of our design, we discussed and compared four counterpart structures, which are the proposed interferometer with bilateral Bragg gratings (I), the interferometer with one Bragg grating (II), the interferometer with bilateral grooves (III) and the interferometer with one groove (IV), as denoted in Figure 5a. Figure 5b depicts the electric field intensity distributions along the upper semiconductor surface for four interferometers and an isolated microslit structure. The parameters used for simulations are *D* = 60 μm, *H* = 300 μm, *T* = 300 K, *N* = 15, *t* = 100 μm, *d*_1_ = 55 μm, *d*_2_ = 50 μm, *L* = 2500 μm, *λ* = 320 μm, *w* = 50 μm and *h* = 100 μm. Compared to the isolated slit, there were obvious interference oscillations on both sides of the slit ranging from −2500 to 2500 μm for all interferometers, suggesting that the reflection of SPP waves takes place when they get to the Bragg gratings and grooves. However, the electric field intensity for interferometers I and II was significantly stronger than those for interferometers III and IV, due to the nearly total reflection by the Bragg gratings (i.e., the electric field intensity transmitted through the Bragg gratings (|x| > 2500 μm) approaches to 0). Figure 5c shows the normalized transmitted intensities for four interferometers when the value of *L* varied from 500 to 2500 μm. It is physically reasonable that the interference oscillations for structures with bilateral Bragg gratings (grooves) were at least two times stronger than those with one grating (groove). Among them, the proposed interferometer shows a remarkable interference pattern contrast, which offers a maximum contrast of 2.14, which was 2 times, 6 times and 14 times higher than those for interferometer II (a maximum contrast of 0.92), interferometer III (a maximum contrast of 0.38) and interferometer IV (a maximum contrast of 0.15), respectively. The results illustrate that the interferometers with bilateral Bragg gratings could offer an enhanced capability for realization of high contrast interference modulation and strong electric field intensity in the vicinity of the semiconductor’s surface.

## 5. Potential for use as Interferometric Sensors

The phase and amplitude of SPPs waves are influenced by the parameters of *n*_d_, which can be respectively modulated by any surface analyte binding, or in other words, the transmitted THz radiation contains the information on the relative phase difference and amplitude of two independent SPPs waves. As a result, it offers a potential for the device to act as a refractive index (RI) sensor by measuring the spectral shift.

**Figure 6:**
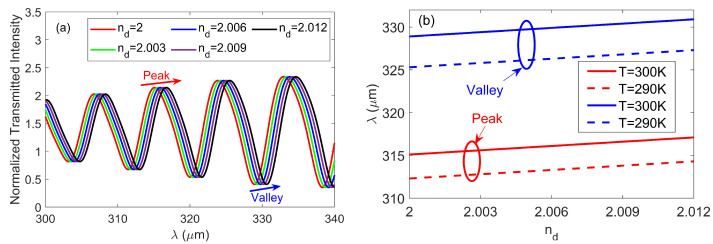
(**a**) Normalized transmitted intensity for proposed interferometers with different surrounding refractive index (RI; *n*_d_) ranging from 2 to 2.012 at room temperature *T* = 300 K. (**b**) Peak (valley) wavelength versus refractive index *n*_d_ with a different operating temperature *T*.

As an example, to verify its potential for biosensing, the yeast was selected as the surrounding analyte, which has a RI of *n*_d_ = 2 [32] in the THz regime. Figure 6a illustrates the dependence of normalized transmitted intensity on the wavelength for different *n*_d_ varying from 2 to 2.012 with a step of 0.003, due to the fact that the variation of RI induced by analyte binding is typically small. The parameters are *D* = 60 μm, *H* = 300 μm, *T* = 300 K, *N* = 15, *t* = 100 μm, *d*_1_ = 55 μm, *d*_2_ = 50 μm and *L* = 2000 μm. As can be seen, the interference pattern shifts towards the longer wavelengths with the increase of *n*_d_ at all incident wavelengths. The fringe shift was around ∆*λ* = 0.5 μm for a RI change of ∆*n*_d_ = 0.003, i.e., the estimated sensitivity (S = ∆*λ*/∆*n*_d_) was 167 μm/RIU, indicating that the variations of *n*_d_ can be monitored by measuring the shift of peak or valley wavelength. The peak and valley positions of the normalized transmitted intensity around 315.1 μm (indicated by the red arrow) and 328.9 μm (indicated by the blue arrow) were measured versus *n*_d_ with different *T* and plotted in Figure 6b. From it one can see that the position of the peaks and valleys had an approximately linear response with the increase of *n*_d_, and they were affected by the temperature, however, the slopes of four lines are identical, indicating that the temperature had an insignificant influence on the sensitivity, which might provide an important promise to elude the ubiquitous problem of cross-sensitivity in multi-parameters sensing cases.

**Figure 7:**
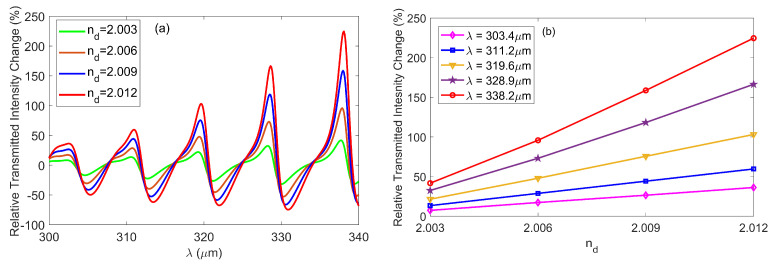
(**a**) Relative transmitted intensity change versus wavelength *λ* for different *n*_d_. (**b**) Maximal value of relative transmitted intensity change as a function of *n*_d_ at a different destructive wavelength.

The proposed interferometer is also more favorable for the intensity-based sensing. For example, as shown in Figure 6a, the destructive interference takes place at the wavelength of *λ* = 328.9 μm where the transmitted intensity was 0.41 when *n*_d_ = 2. However, the value of transmitted intensity was 1.02 at the same wavelength when *n*_d_ = 2.012. Consequently, a little RI variation (∆*n*_d_ = 0.012) will result in an extremely large relative transmitted intensity change ∆T, which was $\Delta T=\left[\left(T_{n_{d}=2.012}-T_{n_{d}=2}\right)/T_{n_{d}=2}\right]\times 100\%$ ≈ 150%. Therefore, the measurement of RI can be achieved by monitoring the value of ∆*T*. Figure 7a shows the spectra of the relative transmitted intensity changes with different value of *n*_d_. When the value of *n*_d_ increases from 2 to 2.012, we can see that the relative transmitted intensity changes reached peak values of 36%, 59.6%, 103.1%, 166.4% and 224.6% at the destructive wavelengths of *λ* = 303.4 μm, *λ* = 311.2 μm, *λ* = 319.6 μm, *λ* = 328.9 μm and *λ* = 338.2 μm, respectively. In Figure 7b, we plotted the relative transmitted intensity change as a function of *n*_d_ at different destructive wavelengths. As expected from Figure 7a, the relative transmitted intensity change got higher as the destructive wavelength increased, and the curves show essentially linear distributions over the entire RI range. The slope of the curve is well-known as FoM, which revealed how steep the relative transmitted intensity change (∆*T*) induced by the RI variation (∆*n*_d_) at a specific wavelength, and is defined by ∆*T*/∆*n*_d_ [33]. It was observed that the maximal FoM was about FoM = 18,750% RIU^−1^ at *λ* = 338.2 μm with *n*_d_ = 2.012.

On the basis of the above discussions, we note that the proposed interferometric sensor could be either operated based on the spectral shift monitoring or the intensity interrogation at several wavelengths, or in other words, it possesses a distinguished spectroscopic capability in practical applications to maintain high sensing performance over a wide wavelength range. Particularly, by comparing the performance with that for the T-shape patterned Interferometers reported in [28], the FoM is moderately enhanced by a factor of 1.2. However, the shortcomings of utilizing the T-shape pattern were also very distinct. For example, its fabrication imperfection was relatively higher, i.e., the uneven surface, which will highly decrease the intensities of the interfering SPPs waves, in turn eventually affected the far-field interference performance.

**Figure 8:**
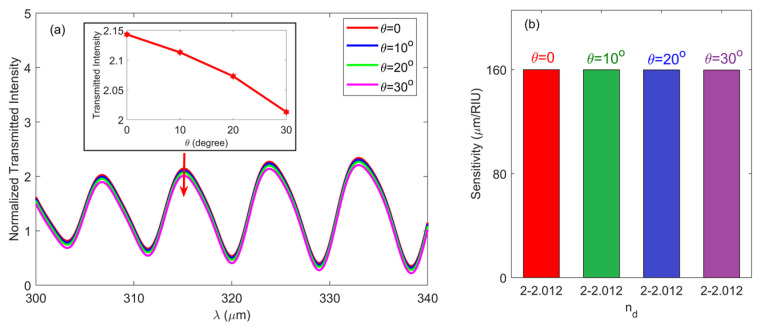
(**a**) Normalized transmission spectra for interferometers with different incident angle *θ* operating at room temperature. Inset: normalized transmitted intensity versus incident angle *θ* at constructive wavelength of 315.1 μm. (**b**) Calculated sensitivities for structures with a different incident angle *θ*.

In addition, it is known that the dimension of the proposed structure was compatible with the spot size of THz wave (i.e., several mm), which could be focused by an off-axis parabolic metal mirror in the THz time-domain spectrometer, indicating that a part of the THz wave will be obliquely incident on the structure in reality [34]. Hence it is important to investigate the influence of the incident angle (*θ*) on the performance of the structure. As an example, Figure 8a depicts the normalized transmitted intensity versus *θ* and *λ* with *D* = 60 μm, *H* = 300 μm, *T* = 300 K, *n*_d_ = 2, *N* = 15, *t* = 100 μm, *d*_1_ = 55 μm, *d*_2_ = 50 μm and *L* = 2000 μm. From it one can see that the locations of resonant wavelengths were slightly affected by *θ*, whereas the transmitted intensity decreased very slowly when *θ* was increased, as shown in the inset of Figure 8a, which depicts the normalized transmitted intensity as a function of *θ* at the constructive wavelength of *λ =* 315.1 μm. For example, as *θ* increased from 0 to 30°, the normalized transmitted intensity only varied by 6%. The reason was because the acceptance incident angle for light coupling into the guided mode inside the microslit cavity was large, which is analogous to that for a single metallic slit [35]. Furthermore, the calculated sensitivities for the four cases are demonstrated in Figure 8b. As clearly seen, the influence of incident angle on the sensitivity was insignificant. Such small variations result from the stable electromagnetic response of the structure for a different incident angle.

**Figure 9:**
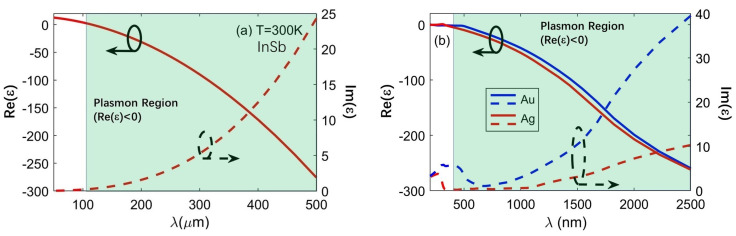
Dependence of real and imaginary parts of permittivity on the wavelength for (**a**) InSb at THz frequencies and (**b**) noble metals (i.e., Au and Ag) at optical frequencies. Light green areas represent cases of Re(*ε*) < 0.

Finally, it is very important to note that the key dimensions of the proposed device (such as *H*, *D*, *d*_1_ and *d*_2_) were in the order of tenths of a micrometer, however, they were still comparable with the operating wavelength. On the other hand, the doped semiconductors at THz frequencies behaved similarly as noble metals working in the visible and near-infrared (NIR) spectral ranges (i.e., approximately from 500 to 2500 nm) [36]. For example, Figure 9a,b shows the dependence of the permittivity (*ε*) on the operating wavelength for InSb at *T* = 300 K and noble metals (i.e., gold and silver), respectively. From Figure 9 one can see that the real and imaginary parts of permittivity (i.e., Re (*ε*) and Im (*ε*)) for InSb and noble metals varied with a similar trend. In particular, the values of Re (*ε*) became negative, or in other words, they could act as plasmonic materials when operated at certain spectral range, as illustrated by the light green area in Figure 9. Therefore, our design also potentially offered the physical insights for helping develop the metallic structures working at optical frequencies with nanoscale dimensions optimized based on Equation (3) and that to maximize the transmission of the central slit [37].

## 6. Conclusions

In summary, we proposed and demonstrated a novel plasmonic interferometric concept that combined plasmonic architectures with interferometry techniques to allow flexible control of the phase and amplitude of interfering SPPs waves in the THz regime. The structure consists of a thin semiconductor layer patterned with a central microslit, which is flanked by two identical Bragg gratings. Two propagating SPP waves were supported on both sides of the microslit, and their phase difference was highly influenced by the variation of the surrounding material. As a result, the THz wave transmitted through the microslit interfered with these two SPPs waves, which were totally reflected by Bragg gratings, giving rise to a strong modulation in the interference spectra with a high-contrast. With the optimized structural characteristics, we have shown that the wavelength sensitivity and FoM based on intensity interrogation could reach up 167 μm RIU^−1^ and 18,750% RIU^−1^, respectively. In addition, we also demonstrated that the proposed sensor has a good tolerance to the incident angle in cases of oblique illumination. It is believed that the proposed new group of interferometry might find important applications involving plasmonic sensing and integrated THz circuits.
